# Supplementary material for: More popular because you’re older? Relative age effect on popularity among adolescents in class
Source: PLoS One. 2021 May 5;16(5):e0249336. doi: 10.1371/journal.pone.0249336 (PMC8099083; doi:10.1371/journal.pone.0249336)
Supplement: S1 Table — (DOCX) [file pone.0249336.s001.docx]

**S1 Table:** **Number of cases and age range per birth month after cut-off date**

|  | EN |  |  | NL |  |  | SW |  |  |
| --- | --- | --- | --- | --- | --- | --- | --- | --- | --- |
| *Birth month after cut-off date* | n | Age-range | | n | Age-range | | n | Age-range | |
|  |  | *Min* | *Max* |  | *Min* | *Max* |  | *Min* | *Max* |
| 0 | 333 | 13.34 | 16.37 | 372 | 14.05 | 17.26 | 352 | 13.07 | 16.05 |
| 1 | 377 | 14.37 | 16.04 | 409 | 13.22 | 17.45 | 347 | 13.22 | 16.05 |
| 2 | 341 | 14.45 | 17.12 | 386 | 13.35 | 16.70 | 357 | 13.25 | 15.46 |
| 3 | 369 | 13.59 | 16.21 | 328 | 13.40 | 16.71 | 390 | 13.25 | 15.51 |
| 4 | 317 | 13.67 | 17.93 | 368 | 14.38 | 16.81 | 375 | 13.60 | 15.61 |
| 5 | 336 | 14.71 | 17.10 | 342 | 13.60 | 16.94 | 466 | 13.54 | 15.61 |
| 6 | 325 | 14.79 | 16.46 | 387 | 13.69 | 17.72 | 424 | 13.57 | 15.77 |
| 7 | 401 | 13.92 | 16.55 | 337 | 13.72 | 17.99 | 416 | 13.57 | 16.76 |
| 8 | 365 | 14.00 | 16.64 | 353 | 13.80 | 18.00 | 462 | 13.75 | 16.80 |
| 9 | 317 | 14.08 | 16.64 | 332 | 13.93 | 17.11 | 448 | 13.77 | 16.02 |
| 10 | 352 | 14.08 | 15.73 | 329 | 13.94 | 17.29 | 407 | 13.84 | 17.30 |
| 11 | 390 | 14.37 | 16.80 | 367 | 13.39 | 17.23 | 419 | 13.08 | 17.00 |
