# Supplementary material for: More popular because you’re older? Relative age effect on popularity among adolescents in class
Source: PLoS One. 2021 May 5;16(5):e0249336. doi: 10.1371/journal.pone.0249336 (PMC8099083; doi:10.1371/journal.pone.0249336)
Supplement: S2 Table — (DOCX) [file pone.0249336.s002.docx]

**S2 Table. Fractional probit estimates of popularity in class**

|  | Full sample | | Netherlands only | |
| --- | --- | --- | --- | --- |
| Variables | Model 1 | Model 2a | Model 2b | Model 3 |
| Country (ref = Netherlands) |  |  |  |  |
| Sweden | -0.039 | 0.061 |  |  |
|  | (0.035) | (0.040) |  |  |
| England | -0.084** | 0.013 |  |  |
|  | (0.037) | (0.042) |  |  |
| Boy | 0.099*** | 0.095*** | 0.104*** | 0.096*** |
|  | (0.015) | (0.015) | (0.027) | (0.027) |
| Class size | -0.033*** | -0.033*** | -0.037*** | -0.038*** |
|  | (0.002) | (0.002) | (0.003) | (0.003) |
| Class popularity (mean) | 0.311*** | 0.312*** | 0.287*** | 0.288*** |
|  | (0.010) | (0.010) | (0.019) | (0.019) |
| % Immigrants in school (ref = 0-10) |  |  |  |  |
| 10-30 | 0.008 | 0.008 | 0.029 | 0.027 |
|  | (0.023) | (0.023) | (0.042) | (0.041) |
| 30-60 | 0.014 | 0.014 | 0.003 | 0.001 |
|  | (0.024) | (0.024) | (0.041) | (0.042) |
| 60-100 | 0.012 | 0.012 | -0.023 | -0.029 |
|  | (0.025) | (0.025) | (0.046) | (0.047) |
| Independent school | 0.043 | 0.041 |  |  |
|  | (0.054) | (0.054) |  |  |
| Past relative age | 0.012*** | 0.005 | 0.006 | 0.009** |
|  | (0.004) | (0.004) | (0.004) | (0.004) |
| Past relative age * Sweden | 0.004 | 0.012* |  |  |
|  | (0.005) | (0.007) |  |  |
| Past relative age * England | 0.014** | 0.020** |  |  |
|  | (0.006) | (0.010) |  |  |
| Current relative age |  | 0.263*** | 0.262*** | 0.125** |
|  |  | (0.047) | (0.047) | (0.061) |
| Current relative age * Sweden |  | -0.285*** |  |  |
|  |  | (0.078) |  |  |
| Current relative age * England |  | -0.247** |  |  |
|  |  | (0.117) |  |  |
| Grade repetition (ref = none) |  |  |  |  |
| Primary |  |  |  | -0.019 |
|  |  |  |  | (0.041) |
| Secondary |  |  |  | 0.338*** |
|  |  |  |  | (0.050) |
| Both |  |  |  | 0.246** |
|  |  |  |  | (0.119) |
| Constant | -1.344*** | -1.440*** | -1.304*** | -1.270*** |
|  | (0.047) | (0.051) | (0.076) | (0.077) |
|  |  |  |  |  |
| Observations | 13,251 | 13,251 | 4,308 | 4,301 |

Robust standard errors in parentheses

*** p<0.01, ** p<0.05, * p<0.1

Note: fractional regression models fit a response model for a dependent variable that is greater than or equal to 0 and less than or equal to 1. For that reason, we rescaled the dependent variable from 0-100 to 0-1. Presented are the results of a probit model for the conditional mean.
