# Supplementary material for: More popular because you’re older? Relative age effect on popularity among adolescents in class
Source: PLoS One. 2021 May 5;16(5):e0249336. doi: 10.1371/journal.pone.0249336 (PMC8099083; doi:10.1371/journal.pone.0249336)
Supplement: S3 Table — (DOCX) [file pone.0249336.s003.docx]

**S3 Table: Three-level random intercept models of popularity in class with interactions of past- and current-relative age and gender**

|  | Model 1 | Model 2 |
| --- | --- | --- |
| Variables |  |  |
| Country (ref = Netherlands) |  |  |
|  |  |  |
| Sweden | -0.235 | -0.366 |
|  | (0.338) | (0.343) |
| England | 0.308 | 0.140 |
|  | (0.334) | (0.343) |
| Boy | 0.333 | 1.525*** |
|  | (0.535) | (0.260) |
| Class size | -0.542*** | -0.541*** |
|  | (0.024) | (0.024) |
| Class popularity (mean) | 5.050*** | 5.047*** |
|  | (0.174) | (0.174) |
| % Immigrants in school (ref = 0-10) |  |  |
| 10-30 | 0.097 | 0.126 |
|  | (0.392) | (0.392) |
| 30-60 | 0.257 | 0.288 |
|  | (0.409) | (0.409) |
| 60-100 | 0.202 | 0.232 |
|  | (0.437) | (0.438) |
| Independent school | 1.932** | 1.989** |
|  | (0.881) | (0.882) |
| Past relative age | 0.235*** | 0.024 |
|  | (0.072) | (0.083) |
| Past relative age * Boy | -0.210** |  |
|  | (0.101) |  |
| Current relative age | 0.676 | 2.030** |
|  | (0.835) | (0.892) |
| Current relative age * Boy | 4.752*** |  |
|  | (1.172) |  |
| Past relative age * Current relative age |  | 0.201 |
|  |  | (0.129) |
| Constant | 9.353*** | 9.243*** |
|  | (0.794) | (0.818) |
| *Number of observations* |  |  |
| Schools (level 3) | 681 | 681 |
| Classrooms (level 2) | 336 | 336 |
| Individuals (level 1) | 13,251 | 13,251 |

Standard errors in parentheses

*** p<0.01, ** p<0.05, * p<0.1
