# Supplementary material for: More popular because you’re older? Relative age effect on popularity among adolescents in class
Source: PLoS One. 2021 May 5;16(5):e0249336. doi: 10.1371/journal.pone.0249336 (PMC8099083; doi:10.1371/journal.pone.0249336)
Supplement: Attachment — Submitted filename: VaAalstTubergen_2020_PlosOne_RAE_Response to Reviewers.docx [file pone.0249336.s004.docx]

**Response to Reviewers**

Reviewer #1: This is a very well written research paper. I really enjoyed reading it. I have just a few minor suggestions for adding some details to make the analysis more meaningful.

1. Can the authors please add a table of age band by month for each country and mention how many cases were in each of these age bands?
*This is a good suggestion. We now include this table in the Supplement (S2 Table)*

2. Can you please add R square value or percentage correctness for each step of the model?
*In this revised paper, we present statistics for model fit. Because we use three-level mixed models (following the suggestion of Reviewer 2, comment 14), we report the Akaike information criterion (AIC), the Bayesian information criterion (BIC), and loglikelihood (LL) for each model presented in Table 3.*

3. So what are the policy implications of these findings? What can teachers learn from this and how can education be improved with this knowledge? Please include this in discussion or conclusion of this article.
*We agree that the policy implications can be more elaborately explained in the discussion**. One policy implication is that teachers in England should become more aware of the enduring impact of the cut-off date when students enter primary school: it affects their well-being, popularity and education outcomes in secondary education. In the Netherlands, by contrast, teachers should realize that grade repetition impacts their students beyond their education outcomes. Those who repeat a grade are more popular than their peers (p. 13).* *Taken together, this study shows that teachers may benefit from becoming aware of the relative age of their students, because it not only impacts students’ educational outcomes, but also their popularity among peers in class -and these two outcomes may be interrelated. (p.14).*

**Reviewer #2:**

The manuscript “More popular because you’re older? Relative age effects on popularity among adolescents in class” uses a large dataset from multiple countries to examine whether students’ past and current relative age is related to students’ popularity status. The large sample size and novel examination of the association between relative age and popularity are strengths of the paper. There are a few methodological and analytic concerns with the manuscript as well as a need for a stronger theoretical foundation for the paper. Specific recommendations for improvement are outlined below.

1. There is a missed opportunity to thoroughly dive into the popularity literature to discuss what popularity is, what it means, what it gives students, and why the features associated with relative age might translate into popularity (e.g., physical size).

*We agree that a more elaborate discussion of popularity was lacking. We added literature on popularity and provided a more in-depth discussion (p. 3)*

2. Is there evidence to suggest popularity is considered the same in those countries? Does it have the same behavioral correlates?

*This is an interesting question. We are not aware of any study which provides evidence that popularity is considered the same across countries, nor that it differs. The data we use were collected in the same way in each country, i.e., pupils were asked the same sociometric questions. In a way to learn more about behavioral correlates, we performed an additional analysis, in which we regressed popularity on survey country, and one well-known behavioral correlate (gender), and interactions. Results are presented in the Supplement (S1 Table), In addition, we performed additional analyses in which we changed the reference category for survey country. Results show that students in Sweden receive fewer popularity nominations on average, as compared with peers in the Netherlands and England. This suggests that the ‘threshold’ for nominating someone as popular in Sweden might be higher. The impact of gender on popularity is very similar across countries.*

3. The authors introduce the new concept of current relative age and while I do believe it has the potential to contribute to our understanding of students’ adjustment in classrooms, a more robust presentation of this theory is needed. Perhaps some discussion of social comparisons and norm group would be helpful. What does the literature on mixed-aged classrooms (e.g., Montessori structure) say about students’ peer relationships and social roles?

*This is an interesting question. We tried to find literature on this, but could not find anything of interest for this paper. We furthermore believe that this concerns such a small group of schools and classrooms in all countries, making us to decide to not go into the relative age effect in mixed classrooms in detail.*

4. The authors indicate that the exact day of birth is known from students in the Netherlands and Sweden but this creates an issue with the relative age variable being created differently for different students depending on their country. I encourage the authors to use the same procedure to calculate relative age using month of birth.

*We agree and therefore adjusted the variable of calculating current relative age, so that we now only include information on month and year of birth for all pupils of all three countries (p.8).*

5. More contextual information is needed about the schools included in the sample. Are these self-contained classrooms where the same group of 14-15 year olds stay with the same group of peers for the entire school day? Or do they switch classrooms (and therefore interact with different peers) for different subjects? Would these students have just transitioned to a new school setting (i.e., secondary/high school)?

*We agree that this information is currently lacking, and needs to be added to provide more insight in the situation of the adolescents in the study.*

*We include more information about the schools and school classes (p.7).* *The data we analyze are from children in self-contained classrooms in third grade (age 14-15). In England pupils enter secondary school at age 11 until they are at least 16.. In the Netherlands, pupils enter secondary education at age 12 and remain in the same self-contained classroom for three years before choosing a path with different subjects. However, even then they still have the same classroom for basic courses such as languages, literature, gymnastics and arts. Finally in Sweden, pupils are in the same classroom during so-called “Grundskola” from age 7 to 16 (European Commission/EACEA/Eurydice, 2018). For the pupils from our sample we can thus safely state that they follow almost all lessons (e.g., languages, literature, mathematics, etc.) with the same group (i.e., self-contained classrooms).*

6. Information on the structure of the secondary schools is needed to judge the peer nomination procedure used in this study. Popularity is typically measured with either a limited number of nominations (i.e., 3) in younger school children (i.e., primary schools) because of the self-contained nature of classrooms, hence the voting population is smaller (Cillessen & Marks, 2017). Once students move to larger schools (e.g., secondary schools), they are less likely to be in self-contained classrooms and to reflect this increase in size in the voting population, unlimited nominations are typically allowed for older students. However, there are important cultural differences in these structures. The authors state that students were allowed to nominate up to 5 classmates. Was this constrained to only peers in their self-contained class? Or were they nominating other students in the school?

*The peers that could be nominated as popular were classmates only. The question that students answered was “**Who are the most popular students in this class? Here you may write down no more than five numbers”. We are aware that at secondary schools, students also have friends outside their classroom, and outside their school, for instance from their sports club or neighborhood. However, we focus here on the popularity hierarchy and age distribution within their self-contained classrooms. From previous research we know that the question and target group that students could choose from are both widely used in other research on social networks of children and adolescents (Dijkstra, Cillessen, and Borch, 2012). We have clarified this measurement in this revised version (p.8)*

7. It was good to see the authors accounted for differences in class size by calculating a proportion score for the popularity nomination measure by dividing each students’ total number of nominations by the number of possible nominators (class size). However, the authors should also standardize the nominations by the voting population unit (e.g., classroom or school) per established peer nomination protocol (Estell, Farmer, & Cairns, 2003; Rodkin, Farmer, Pearl, & Van Acker, 2000) to account for the fact that classrooms will have different nomination averages for popularity.

*We would like to thank the reviewer for this suggestion. We agree that it is important to standardize as much as possible to be able to generalize findings. We therefore created an additional variable that calculates the average popularity per country per class. We added this variable as control variable in the main analysis (see also text at p.10) and the results can be found in Table 3.*

8. The authors provide the participation rates for the schools but equally important for the peer nomination measure is the participation rate for classrooms where popularity nominations were gathered. Marks, Babcock, Cillessen, and Crick (2013) recommend that a certain participation rate threshold be met (e.g., at least 40% for very salient nominations, otherwise 60%).

*Per school, two or three classrooms were randomly selected by the research team. All of these pupils in these two classrooms were then approached by the researchers. The participation rate for pupils was high (>80% in England, >85% in Sweden, >90% in the Netherlands). We checked the data to see if there were any classrooms with participation rates below 60%, but this was not the case (see for more details the Fieldwork Report (Kruse, H., & Konstanze, J. (2016). Children of Immigrants Longitudinal Survey in Four European Countries. Sociometric Fieldwork Report. Wave 1 – 2010/2011, v1.2.0.) (we also refer to this report on p.7).*

9. Can the author provide the exact language used for the peer nomination measure for popularity in each country? Was the nomination based on free recall or were students provided with class lists?

*The question asked to students was similar in all three countries, and nominations were based on provided class lists where each student was assigned a random number. Students were asked to write down the numbers corresponding to the student in answering the questions.*

*EN: Who are the most popular students in this class? Here you may write down no more than five numbers
NL: Wie zijn de populairste leerlingen van de klas? Je mag hier maximaal 5 nummers opschrijven.
SW: Vilka är de mest populära eleverna i klassen? Här ska du inte skriva ned fler än fem nummer.
In all three countries there was the option to nominate no classmates at all, we also mentioned this in the method section (p.8).*

10. Information on how data was collected is missing and a brief summary of the data collection procedures is needed. Did students fill out pen and paper surveys or was data collected electronically? When was data collected during the school year?

*We agree that we could provide some more information about the data collection and its implications for the results. Data collection consisted of different parts, with an interview to test cognitive and language abilities, and a 45-minute self-reported questionnaire filled in by students with paper and pencil, which we also added in the text (p.7). For detailed information we refer to the codebook and technical report of CILS4EU that can be found online (p.7).*

11. The authors state a final sample of 13,014 students but I could not find information about the size of the initial sample and how the initial versus final sample differed.

*The full CILS4EU dataset included 18,716 pupils. However, Germany (N = 5,013) was excluded from our study, because the cut-off date could not be clearly defined. From the resulting sample (13,703) we removed only 3% (452 pupils): 75 pupils were removed as they had missing values on birth month, 71 pupils missed data on year of birth which we used to calculate current relative age, 9 pupils had a missing value on sex, and 297 Swedish pupils were removed who repeated a class. The final sample size was 13,251. (p.7)*

12. I recommend the authors consider including two important interaction effects in their analyses. First, I encourage the authors to examine the interaction between past and current relative age to better understand whether being both historically older (past age) and being oldest in the class currently is associated with higher population (i.e., does being currently older strengthen the association between past relative age and popularity?) or does current relative age not matter as much as being older from the start (past relative age)?

*We think this is a very interesting interaction to test. Results are now included in S4 Table and discussed in the paper (p.12).*

13. Another interaction I was surprised to see not considered was sex differences. Developmentally, 14-15 year olds are likely to be at various stages in pubertal development which will impact characteristics and features often associated with popularity such as physical size, attractiveness, etc. Is the association between relative age and popularity stronger for girl or boys? I strongly recommend the authors consider the role of gender in their analyses.

*This is also an interesting idea. We added the findings to S4 Table (and see p.12 for discussion).*

14. One major concern with the analyses is that the nested nature of the data was not accounted for. The authors should strongly consider multilevel analyses and provide a rationale for their analytic decision.

*We agree and now present results from multilevel analyses, i.e., we estimate three-level random intercept models (level 3: schools, level 2: classrooms, level 1: pupils). (we also mention this at p.10)*

15. The authors are trying to examine whether different education policies at the country level impact the association between relative age and popularity. They report that 5% of the students in Sweden had repeated a grade, even though they have a policy of social promotion. Were these 5% of students removed from the analyses? If not, this would be problematic because it represents a confound in the analyses between the Netherlands and Sweden. If this subgroup of students were removed, does that change the authors’ expectation that Sweden would fall between England and the Netherlands?

*We agree that it is indeed blurring the results if we would include these Swedish students who repeated a grade. In order to keep the analysis as close to the policy and thus reality, as possible, we decided to remove the Swedish pupils who repeated a grade (N=297, 5%). We also mention this in the text at the end of the method section (p.7)*

16. Because of the focus on education policy in the paper, I encourage the authors to provide a more robust discussion of how exactly the results have implications for policy. Is one policy “better” than the other? How do they define what “better” means in terms of these associations with popularity? What are the implications for students’ social development depending on each policy?

*We agree that it is interesting to elaborate a bit more in the discussion section (p.13).*

17. One limitation to acknowledge in the discussion section is that the study cannot speak to the mechanisms driving the association between relative age and popularity. It is unlikely to be age per se, but the associated characteristics, skills, or developmental milestones that come with being older among younger peers.

*We agree and discuss this issue in the revised paper (p.14).*
