# Supplementary material for: More popular because you’re older? Relative age effect on popularity among adolescents in class
Source: PLoS One. 2021 May 5;16(5):e0249336. doi: 10.1371/journal.pone.0249336 (PMC8099083; doi:10.1371/journal.pone.0249336)
Supplement: Attachment — Submitted filename: Response Letter_12mrt21.docx [file pone.0249336.s005.docx]

**Response Letter**

Dear editors,

Attached please find the revised version of manuscript *“More Popular Because You’re Older? Relative Age Effect on Popularity among Adolescents in Class*.” We would like to thank you for the detailed reading of our manuscript and the constructive feedback. We made every effort to address each point raised in the revised manuscript and in the present letter. We hope the manuscript now meets the standards for publication in *PlosOne.*

Best wishes,

Danelien van Aalst and Frank van Tubergen

| **Comments** | **Answer** |
| --- | --- |
| To improve the flow and organization of this paragraph, I recommend moving the two sentences on p. 3 starting with “Popularity is defined as…” to after the sentence “Popularity and social preference can thus be seen as…” At present, the discussion goes from specifics about popularity, more broadly to types of social status, then back again to specifics about popularity. I would streamline this by starting with the broad discussion of social status and how it’s important to youth then narrowing the focus to the definition of popularity. | We have moved these two sentences to the end of the paragraph (p.3) |
| I appreciated your attempt to respond to my previous comment about behavioral correlates of popularity in these countries by looking at gender difference but gender should not be referred to as a behavioral correlate. Additionally, the intent behind my original comment was to have you review any available evidence of associations between popularity and behavior/ peer experiences (e.g., bullying, victimization) using adolescent samples from those countries to address whether popularity is seen similarly across country contexts. I know of several such studies from the Netherlands (e.g., Cillessen et al., 2014; de Bruyn, Cillessen, & Wissink, 2010) but cannot immediately recall studies using English or Swedish adolescent samples. Perhaps there are some looking at popularity and bullying/ victimization that you can identify. In the absence of any available evidence, please include a discussion in the limitation/ future direction section that more research is needed to verify whether popularity is associated with similar characteristics and behavior in these countries. | We have removed the additional analysis, in which we regressed popularity on survey country, gender and interactions (Supplement S1 Table).  We now understand that you referred to bullying and victimization as behavioral correlates, and that the question is whether these are similarly related to popularity in all three countries. We thank you for the suggested references for the Netherlands, and included these in this part, and also some studies that found the same association between popularity and bullying in other countries, strengthening our assumption that the sociometric measure for popularity has a similar meaning in all three countries. |
| Clearer statements in your conclusion are needed that describe the results in terms of youth’s popularity status to aid in readers’ interpretation of the findings. Something like: “Youth who were older (i.e., past relative age) when they started school are more likely to be seen as popular when they are in secondary school.” | We added this sentence to explain better what the results of the study were: “In other words, youth who were relatively older when starting primary school were more likely to be seen as popular in secondary school.” (p.13) |
| Please temper the causal language in the discussion (e.g., impact). | We substituted causal language by referring to the relation with popularity (p.14). |
| Please include citations to support statements in your conclusion (e.g., that cut-off dates impact students’ well-being and educational outcomes; that physiological differences include dynamics of competition and status among peers, etc). | We have now included citations in the conclusion as well. |
| You mention in the discussion about how class repetition does occur in Sweden sometimes. However, based on the methods (and in response to my previous feedback), you removed these youth who repeated a class from the analyses. Therefore, the results for the Swedish students included in the analyses cannot be explained by the possibility of grade repetition. How else could you interpret this? | You are correct that we removed the pupils in Sweden who reported that they repeated a grade. However, this measure may not capture all pupils in our dataset who repeated or skipped a grade in Sweden. First, because ‘grade repetition’ is not in line with the official policy of social promotion. Hence, it may occur more widely than we find (5%); it may be something that happens in practice but without the ‘label’ of ‘grade retention’ associated with it. There can also be pupils in Sweden who were kept one year longer in kindergarten, and they may not report that they have repeated a grade. Finally, the data we use did not measure class skipping, so we could not remove those who skipped a class from the data. We address these issues in the revised paper. |
| Please include more discussion about why Sweden and Netherlands did not differ in the association between past relative age and popularity given their different education policies. What could explain their similarity? Are there cultural differences between England versus Sweden and Netherlands that could explain popularity dynamics as it relates to relative age? | We include a discussion of the similarity between Sweden and the Netherlands in the conclusion. The strong contrast is that between UK and the Netherland -Sweden is in the middle between these two: in theory it has a system of social promotion (like UK), but in practice, we see that grade retention occurs (like the NL), but not so often. |
| A more robust discussion is needed for how the association between current relative age and popularity was dependent on the context: Netherland, with its grade repetition policy, may have classrooms with wider range of students, amplifying the (potential) visible differences between youth and contributing to popularity nominations (indeed, a quick visual search of S2 table shows more age ranges spanning 13-17 years). | This is a very good remark. Indeed, the age differences are larger in Dutch classrooms, which makes current age a more salient category. We included a more robust discussion in this revise. |
| Please include a discussion to help readers interpret the gender differences found and reported in S4 Table: that the positive association between past relative age and popularity was stronger for girls whereas the associations between current relative age and popularity was stronger for boys. What could explain these findings? | We have no explanation for this gender difference. It is a finding we reported in the Supplement, and we feel that it would go beyond the scope of the paper to explain such gender differences. But, because we also feel that it is an important discovery, we keep the table in the paper and encourage follow-up studies to explain it. |
| Minor changes: |  |
| p. 3: change “having influence” to “have influence” | We adjusted this (p.3) |
| p. 3: Address confusion with the phrase: “in a highly developmental stage.” Do you mean an age when differences in physical development and maturation are more evident? I see that you do later on in the sentence so my recommendation would be to simply remove the phrase “adolescents are in a highly developmental stage” | We removed this part of the sentence (p.3) |
| p. 4: Change the phrasing at the end of the first paragraph to reflect that these statements are expectations. For instance, instead of “they are more likely to be…” perhaps “it’s reasonable to expect them to be…” or “they may be more likely to be perceived as…”. | We adjusted this into “It is reasonable to expect that these adolescents are more likely to be perceived as powerful, influential and, more self-confident.” (p.3) |
| p. 11: Change “has” to “had” and “has been” to “was” – “An ethical review of…had not been carried out prior to data collection…the CILS4EU project was submitted to the Ethic Committee…” | We adjusted this (p.7 and p.11) |
| p. 13: I would not refer to gender as a behavioral correlate of popularity (see comment above). | We removed this sentence and added information on the behavioral correlate bullying in relation to popularity in all three countries (see our answer to your previous comment) |
| p. 16: instead of “effect” use “association” to reflect the cross-sectional nature of the study. | We checked the whole document on the use of causal language, and substituted this with the words association or relation to make clear we make statements about cross-sectional data. |
| p. 28: Table 2. Please remove the Min and Max values for dichotomous variables (country, gender, etc). You may need to reconfigure the table to have a section for continuous variables to report Ms and SDs and another section to report % of the characteristics at each level. | We collapsed the two columns (Min and Max) into a new column called Range. Following journal conventions, we then use 0/1 to indicate dummy variables, and 0-1 for proportions. |
| p. 27-34: Please make sure only the final tables remain in the manuscript in the next submission. | We only added the relevant tables 1-3 in the manuscript |
| p. 21 Table 1: England has the superscript a next to it. Please inform readers what this means in the Note for the table. | We removed this superscript in the most recent version |
